# Does high-density lipoprotein influence the development of saphenous vein graft disease after coronary bypass surgery?: exploratory analysis from the CASCADE trial

**DOI:** 10.1186/1749-8090-8-172

**Published:** 2013-07-10

**Authors:** Katie Jerzewski, Marc Ruel, Pierre Voisine, Michel R Le May, Alexander Kulik

**Affiliations:** 1 Lynn Heart and Vascular Institute, Boca Raton Regional Hospital, 801 Meadows Road, Suite 104, Boca Raton, Florida 33486, USA; 2 The University of Ottawa Heart Institute, Ottawa, Ontario, Canada; 3 Hôpital Laval, Quebec City, Quebec, Canada; 4 Charles E. Schmidt College of Medicine, Florida Atlantic University, Boca Raton, Florida, USA

**Keywords:** Coronary artery bypass graft surgery, Vein graft disease, HDL, Graft patency, Cholesterol

## Abstract

**Background:**

Low levels of high-density lipoprotein (HDL) purportedly increase the risk after coronary bypass surgery. This may relate to the development of saphenous vein graft (SVG) disease early postoperatively, but this premise has never been evaluated in the context of a prospective trial.

**Methods:**

The CASCADE Trial was a multi-center study of 113 patients evaluating the use of postoperative clopidogrel. Patients received standard lipid management after surgery (96% statins). At 12 months, angiography and intravascular ultrasound was performed to assess SVG occlusion and intimal hyperplasia, respectively. In this exploratory analysis, we evaluated the influence of HDL levels on the development of SVG disease at 12 months, using the established cut-off of <40 mg/dL suggesting increased risk.

**Results:**

While HDL levels increased over the time-period of the trial (P < 0.0001), 51.1% of patients had HDL levels <40 mg/dL 12 months after surgery. Slightly more SVG occlusions occurred amongst patients with HDL levels <40 mg/dL (6.8%), compared to patients with HDL levels >40 mg/dL (4.0%, P = 0.5). With multivariate adjustment, HDL level <40 mg/dL was associated with a trend towards more SVG occlusions (odds ratio: 3.2; P = 0.12). Lower HDL level was also associated with more intimal hyperplasia on ultrasound at 12 months (P = 0.10). Patients who had HDL levels >60 mg/dL had the least amount of intimal hyperplasia, significantly less than the remainder of the cohort (P = 0.01).

**Conclusions:**

Within this population, lower HDL levels were associated with trends towards more graft occlusions and more vein intimal hyperplasia. Modulation of postoperative HDL levels may represent a valuable future strategy for the reduction of SVG disease.

## Background

The saphenous vein remains the most commonly utilized bypass conduit during coronary artery bypass graft surgery (CABG) due to its ready availability and ease of use. The long-term durability of this conduit, however, is limited by the development of saphenous vein graft (SVG) disease during the months and years after surgery [1]. The process of SVG disease, in the form of intimal hyperplasia and atherosclerosis, is strongly influenced by hyperlipidemia [1,2]. Much focus has therefore been directed towards the reduction of low density lipoprotein (LDL) cholesterol levels as a means of improving graft patency [2,3], with current guidelines recommending statin treatment for all patients after CABG to achieve LDL levels <100 mg/dL [4]. Nevertheless, many patients remain at high risk for adverse events even when their LDL levels have been aggressively reduced by statins [5,6].

As such, increasing attention in the cardiology community has recently been directed to the evaluation of therapies to raise high density lipoprotein (HDL) levels to further improve cardiovascular outcomes [5,7-9]. Low HDL levels are commonly seen in patients with coronary artery disease (CAD) and have been well-described as an independent risk factor for adverse outcomes in several studies [5,6,10,11]. Less is known regarding the relevance of HDL after CABG [12,13]. Data from the pre-statin era suggest that HDL can influence the development of SVG disease in the years that follow surgery [14]. While the mechanism is unclear, higher levels of HDL particles may slow the process of SVG disease by preventing atherogenesis, reducing vascular inflammation and improving endothelial function [1,6]. To our knowledge, no study to date has evaluated whether HDL levels have an impact on the early development of SVG disease within the first year after surgery. Using angiographic and intravascular ultrasound (IVUS) data collected as part of the recent Clopidogrel after Surgery for Coronary Artery Disease (CASCADE) clinical trial [15,16], the purpose of this post-hoc study was to explore the influence of HDL on the development of SVG disease early after CABG within a contemporary cohort of patients aggressively treated with preventative therapies.

## Methods

### CASCADE trial design

The CASCADE Trial was a dual-center randomized placebo-controlled trial that evaluated the addition of clopidogrel to aspirin on the development of SVG disease following CABG. Details of the study design and eligibility criteria have previously been published [15]. In brief, 113 patients who underwent CABG using at least two SVGs were enrolled in the trial. The Human Research Ethics Boards of the University of Ottawa Heart Institute and Hôpital Laval approved the study protocol, and each patient provided written informed consent before enrollment. During the operation, the size of each target vessel was noted, and the quality of each target vessel was rated subjectively by the surgeon (good, fair, or poor). Saphenous vein was harvested using open techniques in the CASCADE trial, and endoscopic methods were not employed. Following surgery, patients were randomized to receive either daily aspirin 162 mg plus clopidogrel 75 mg or aspirin 162 mg plus placebo, starting on the day of surgery, for the duration of 12 months.

Twelve months after CABG, patients underwent angiography of the bypass grafts and the native coronary arteries. During the same session, intimal hyperplasia of one randomly selected SVG was assessed by IVUS [15]. A 40 MHz IVUS catheter (Atlantis SR Pro, Boston Scientific Corporation, Natick, Massachusetts, USA) was used to analyze the most proximal 40 mm of the selected graft. The lumen and external elastic lamina area were measured for 1 mm cross sections, and the area of intimal hyperplasia was determined. Mean intimal area per patient was calculated for the 40 mm-analyzed segment.

Twelve-month angiography was completed in 92 of the 113 enrolled patients (81.4%). Intravascular ultrasound was performed in 90 of the 92 patients (97.8%) who underwent follow-up angiography. Intravascular ultrasound could not be performed in 2 patients due to technical factors (1 patient) or the presence of 2 occluded SVGs (1 patient). As detailed elsewhere, the addition of clopidogrel to aspirin did not significantly reduce the process of SVG intimal hyperplasia or result in improved vein graft patency 12 months after CABG [16].

### Patient follow-up

While enrolled in the CASCADE Trial, patients were followed with clinic visits at 1, 6 and 12 months after surgery and telephone home assessments at 3 and 9 months. All patients were treated in accordance with current guidelines [4]. This included smoking cessation counseling, aggressive diabetes management, and the administration of aspirin, beta-blockers, angiotensin converting enzyme inhibitors, and statin medications. Patients were subjected to the standard of care with respect to lipid therapy after surgery. In the absence of an absolute contraindication, surgeons and cardiologists were advised to administer statin therapy postoperatively to each patient enrolled in the CASCADE Trial as recommended in the current guidelines [4]. The statin type and dose was left to the discretion of the prescribing physician and was not dictated by the trial protocol. Lipid levels, including HDL, were assessed prior to surgery, and after surgery at 1, 6, and 12 months. Modulation of HDL levels with medical therapy (i.e. niacin) was not endorsed as part of trial enrollment, given the absence of evidence [7] or guideline statements [4] supporting this practice. Office or telephone assessments were performed every 3 months to ensure medication compliance, including statin therapy. For the purpose of the present study, “consistent statin use” was defined as the preoperative and continued postoperative use of statin therapy throughout the 12-month period of the CASCADE Trial, reported by 62.8% of patients (71/113). Twelve-month clinical follow-up was complete for all patients.

### Statistical analysis

In this exploratory analysis of the CASCADE Trial, we assessed the influence of HDL levels on 12-month graft occlusions and vein intimal hyperplasia. Standard descriptive statistical analyses were used, including repeated measure analysis of variance to examine HDL levels over time. To evaluate the impact of HDL levels following CABG, graft occlusions and SVG intimal hyperplasia were compared between patients who had HDL levels <40 mg/dL, 40–60 and >60 mg/dL, as measured 12 months after surgery. Continuous data are presented as a mean ± standard deviation and were compared between groups using unpaired two-sided Student’s t tests. Categorical data are presented as proportions and were compared between groups using a Fisher’s exact test. Multivariate logistic regression analysis was used to identify independent predictors of vein graft occlusion, and linear regression analysis was performed to evaluate predictors of SVG intimal hyperplasia. The following factors were considered in the multivariate models: preoperative HDL level, HDL level 12 months after surgery, preoperative LDL level, LDL level 12 months after surgery, consistent statin use, age, female gender, body mass index, diabetes, smoking, off-pump surgery, and surgical center. Stepwise forward selection and backward elimination techniques were employed with P = 0.20 for entry and removal criteria. Odds ratios (OR) are reported for the logistic analysis, and regression coefficients are reported for the linear analysis ± standard error. All reported P values are two-sided. P values <0.05 were considered significant, and P values between 0.05 and 0.20 were considered trends towards statistical significance. Data were analyzed in Intercooled Stata 11.0 (Stata; College Station, Texas, USA).

## Results

### Patient cohort

The CASCADE Trial cohort consisted of 113 patients who underwent CABG between 2006 and 2009. The mean age of the cohort was 66.5 ± 7.6 years, and 101 patients (89.4%) were male (Table 1). Most patients had been diagnosed with hypertension (77.9%) or hyperlipidemia (87.6%) prior to surgery. Ten surgeons at 2 surgical centers performed a mean of 3.5 ± 0.7 grafts (range 2–6 grafts; total 396 grafts), with the majority of patients undergoing on-pump surgery. All patients but 1 received a left internal thoracic artery graft. The mean hospital length of stay after surgery was 8.7 ± 5.8 days. Most patients were treated with statin therapy before surgery (89.4%), and nearly all patients received statin therapy after CABG (96% at 12 months).

**Table 1:** Patient characteristics

	**N = 113**
Characteristic	
Age, years	66.5 ± 7.6
Male gender	101 (89.4%)
Body-mass index, kg/m^2^	28.4 ± 3.9
Diabetes mellitus	33 (29.2%)
Current smoker	15 (13.3%)
Hypertension	88 (77.9%)
Hyperlipidemia	99 (87.6%)
Acute coronary syndrome*	21 (18.6%)
Heart failure NYHA class 3-4	23 (20.4%)
Preoperative laboratory values	
Baseline high-density lipoprotein, mg/dL	38.4 ± 11.2
Baseline low-density lipoprotein, mg/dL	72.8 ± 30.0
Baseline creatinine, μmol/L	89.0 ± 17.5
Preoperative medication use	
Aspirin	104 (92.0%)
Clopidogrel	12 (10.6%)
Statin	101 (89.4%)
Beta-blocker	87 (77.0%)
Angiotensin converting enzyme	
Inhibitor	57 (50.4%)
Postoperative medication use (at discharge)	
Aspirin	113 (100%)
Clopidogrel	56 (49.6%)
Statin	103 (91.2%)
Beta-blocker	105 (92.9%)
Angiotensin converting enzyme	
Inhibitor	40 (35.4%)
Operative details	
Number of distal anastomoses	3.5 ± 0.7
Left internal thoracic graft	112 (99.1%)
Right internal thoracic graft	22 (19.5%)
Cross-clamp time, min	64.5 ± 20.1
Cardiopulmonary bypass time, min	90.1 ± 24.6
Off-pump surgery	4 (3.5%)
Length of stay	
Duration in intensive care, days	1.4 ± 1.0
Duration in hospital, days	8.7 ± 5.8

*NYHA* New York Heart Association.

*Recent acute coronary syndrome within 1 month.

### HDL levels

**Figure 1:**
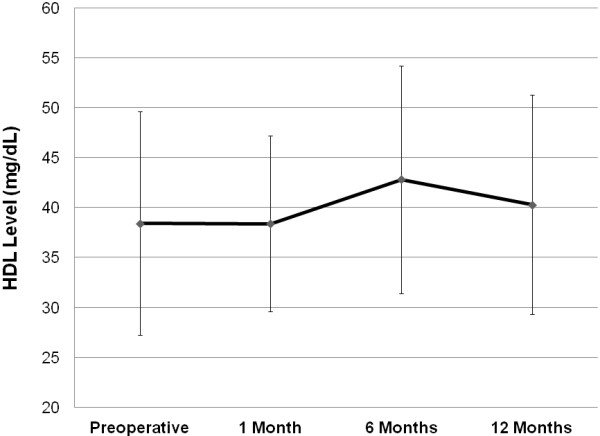
**High-density lipoprotein level during trial enrollment. **High-density level significantly increased during the time period of the trial (P < 0.0001).

Before surgery, 54.1% of patients had HDL levels <40 mg/dL, 45.0% of patients had HDL levels 40–60 mg/dL, and 0.9% of patients had optimal HDL levels >60 mg/dL. Compared to preoperative values, HDL levels significantly increased (P < 0.0001 by repeated measure analysis of variance) over the time period of the study (Figure 1). Whereas preoperative HDL levels were 38.4 ± 11.2 mg/dL, at 12 months after surgery, HDL levels were 40.3 ± 11.1 mg/dL (preoperative versus 12 months postoperative, P = 0.03). Twelve months after surgery, 51.1% of patients had HDL levels <40 mg/dL, 42.2% of patients had HDL levels 40–60 mg/dL, whereas only 6.7% of patients had optimal HDL levels >60 mg/dL. Patients who had HDL levels <40 mg/dL at 12 months had significantly lower LDL levels before surgery compared to patients who had HDL levels >40 mg/dL (preoperative LDL: 66.2 ± 22.8 mg/dL versus 84.4 ± 38.3 mg/dL, HDL level <40 mg/dL versus HDL level >40 mg/dL, P = 0.008).

### HDL and graft occlusion

The twelve-month graft occlusion rate in the CASCADE Trial was 15 of 322 examined grafts (4.7%). The twelve-month internal thoracic artery graft occlusion rate was 1.8% (2/112), and the 12-month SVG occlusion rate was 6.2% (13/209).

**Figure 2:**
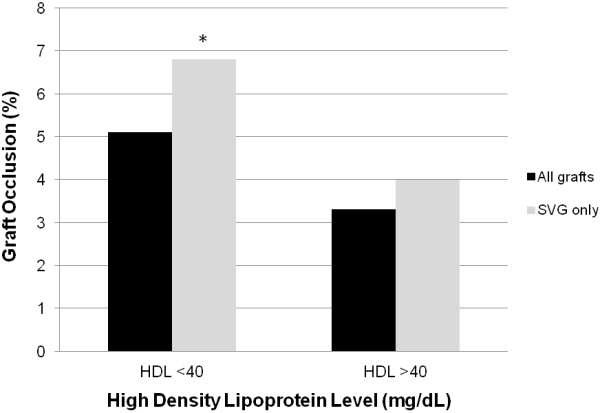
**Impact of high-density lipoprotein level on 12-month graft occlusions. ** Patients with high-density lipoprotein (HDL) levels <40 mg/dL had a trend towards more vein graft occlusions 12 months after surgery. * P = 0.12 compared to HDL level >40 mg/dL in multivariate analysis. HDL, high-density lipoprotein; SVG, saphenous vein graft.

For patients who had HDL levels >40 mg/dL 12 months after surgery, the overall graft occlusion rate was 3.3% (5/152), and the SVG occlusion rate was 4.0% (4/99). In contrast, for patients with HDL levels <40 mg/dL, the overall graft occlusion rate was slightly higher at 5.1% (8/158, P = 0.57), and the SVG occlusion rate was higher at 6.8% (7/103, P = 0.54). However, statistical significance was not reached (Figure 2). Increases in HDL levels to >60 mg/dL (6 patients) or decreases to <30 mg/dL (13 patients) did not significantly impact the SVG graft occlusion rate [P = not significant (NS)]. Preoperative HDL level was not associated with a change in the graft occlusion rate (P = NS).

Multivariate logistic regression analysis was used to identify factors independently associated with SVG occlusions 12 months following CABG. In the multivariable model, 12-month HDL level <40 mg/dL was associated with a trend towards more SVG occlusions (OR: 3.2; P = 0.12). LDL level >100 mg/dL was associated with more occlusions in the model (OR: 14.7; P = 0.02), whereas good target vessel quality was associated with less occlusions (OR: 0.3; P = 0.06). Preoperative HDL level <40 mg/dL or increases in postoperative HDL to >60 mg/dL were not significant factors in the multivariate model (P = NS).

### HDL and intimal hyperplasia

**Figure 3:**
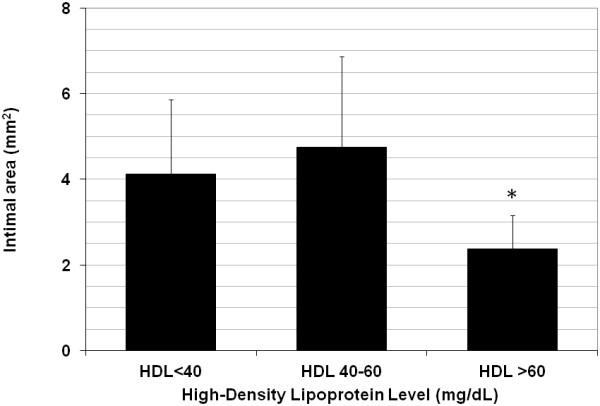
**Impact of high-density lipoprotein level on 12-month vein graft intimal hyperplasia. **Patients with high-density lipoprotein (HDL) levels >60 mg/dL had significantly less intimal hyperplasia 12 months after surgery. * P = 0.01 compared to HDL level <60 mg/dL. HDL, high-density lipoprotein.

The mean 12-month SVG intimal area in the CASCADE Trial was 4.31 ± 2.06 mm^2^, with considerable variation in intimal hyperplasia amongst the enrolled patients (95% confidence interval: 1.89, 7.97 mm^2^). Lower HDL level was associated with a trend towards more intimal hyperplasia on IVUS 12 months after surgery (0.92 ± 0.73 mm^2^, P = 0.2). For patients who had HDL levels <40 mg/dL 12 months after surgery, intimal area was 4.42 ± 2.14 mm^2^, compared to 4.12 ± 1.73 mm^2^ for patients who had HDL levels >40 mg/dL (P = 0.5). Increasing HDL level from <40 mg/dL to 40–60 mg/dL (4.75 ± 2.12 mm^2^), did not significantly reduce intimal area at 1 year (P = NS), and decreases to <30 mg/dL also did not impact intimal area (4.68 ± 1.78 mm^2^, P = 0.4). However, patients who had HDL levels >60 mg/dL 12 months after surgery had the least amount of intimal hyperplasia on IVUS (Figure 3). Compared to patients who had HDL levels <60 mg/dL (4.41 ± 1.93 mm^2^), patients with HDL levels >60 mg/dL had significantly lower intimal area (2.38 ± 0.77 mm^2^, P = 0.01).

In multivariate linear regression analysis, lower HDL level was associated with a trend towards more intimal hyperplasia 12 months after surgery (1.23 ± 0.74 mm^2^, P = 0.10). In the model, older age was independently associated with more intimal hyperplasia (0.06 ± 0.03 mm^2^ per each additional year, P = 0.05), whereas diabetes was associated with a reduction in SVG intimal hyperplasia (-1.02 ± 0.45 mm^2^, P = 0.03). Consistent statin use throughout the trial and LDL level did not significantly predict intimal area in the multivariate analysis (P = NS).

## Discussion

Strong evidence is available to support the use of statins to reduce LDL levels and improve the outcomes of patients recovering from CABG [2-4]. Less is known, however, regarding the influence of HDL on the outcomes of patients after CABG. Previous research has suggested that a relationship exists between lower HDL levels and higher risk of atherosclerosis progression and adverse events following surgery [12-14]. Nevertheless, these studies were conducted in an earlier era, before the routine use of postoperative statins and antiplatelet agents. With a growing interest in the study of HDL therapies in the cardiology community [5,7-9], we sought to evaluate the relevance of HDL levels on the process of SVG disease within a cohort of contemporary CABG patients who were treated aggressively with secondary preventative therapies.

In this exploratory analysis of the CASCADE trial, we assessed graft occlusion rates and vein intimal hyperplasia 12 months after CABG, comparing the degree of SVG disease between patients with low HDL levels to those with higher and more ideal levels. Using an established cut-off of increased risk (<40 mg/dL) [10,11], we noted slightly more SVG occlusions amongst patients with HDL levels <40 mg/dL (6.8%) compared to those with HDL levels >40 mg/dL (4.0%), although statistical significance was not seen (P = 0.54). After adjustment in multivariate analysis, HDL level <40 mg/dL was associated with a trend towards more SVG occlusions (OR: 3.2; P = 0.12). Lower HDL level was also associated with a trend towards more intimal hyperplasia on IVUS. Interestingly, patients with ideal levels of HDL >60 mg/dL had the least amount of intimal hyperplasia, significantly less compared to the rest of the cohort (P = 0.01).

Lower levels of HDL are well-known to be associated with worse clinical outcomes amongst CAD patients [5,6,10,11]. Some of the earliest data on the subject became available from the Framingham Heart Study, whereby low HDL was found to be a more potent CAD risk factor than high LDL [10]. Recent studies from the current era have shown that HDL levels are inversely related to cardiovascular events, even amongst patients receiving statin therapy and those with LDL levels aggressively treated to <70 mg/dL [5,11]. In addition, moderate increases in HDL appear to be associated with regression of coronary atherosclerosis [17].

Given the risk for adverse events that remains despite statin treatment [5,6], several research groups have focused their attention on the evaluation of therapies to increase HDL and thereby improve clinical outcomes in patents already treated with preventative medications [7-9,18]. A number of pharmacological interventions have been shown to improve HDL levels in clinical trials, including niacin [7,8], gemfibrozil [14], bezafibrate [19], fenofibrate [20], and torcetrapib [18]. While some studies have demonstrated modest biological effects, such as the reduction of either carotid artery intimal thickness [8] or angiographic CAD progression [14,19,20], the majority of the studies in the field have produced negative clinical results [7,18]. Casting doubt on the HDL theory, no therapy to date has been shown to increase HDL levels and improve outcomes in a clinical trial enrolling CAD patients already treated with statins [7,18].

In contrast to the attention that HDL has captured in the cardiology community, few studies have evaluated the influence of HDL levels on the outcomes of patients following cardiac surgery. Previous work from the Cleveland Clinic has demonstrated associations between lower HDL and both worse long-term survival and higher risk of adverse events after CABG [12,13]. In the only prospective randomized study in the field, the Lopid Coronary Angiography Trial (LOCAT) enrolled 395 men with HDL levels <42.5 mg/dL who had undergone CABG on average 2 years earlier. From an era prior to the routine use of statins after CABG, patients were randomized to receive either slow-release gemfibrozil 1200 mg per day or matching placebo. Coronary angiography was performed at baseline and after a mean of 32 months of therapy. Gemfibrozil therapy led to significant increases in HDL levels (P < 0.001) and slowed the progression of native CAD (P = 0.009). Moreover, gemfibrozil significantly reduced the risk of developing new lesions in bypass grafts on follow-up angiography (2% versus 14%, gemfibrozil versus placebo, P < 0.001) [14]. Despite the notable results of LOCAT, gemfibrozil never became incorporated into the routine care of CABG patients. This is likely a reflection of the impressive data published that same year promoting the use of statins after CABG [2], as well as the high risk of side effects associated with gemfibrozil when combined with statin therapy [21].

Similar to LOCAT, our exploratory study provides support, albeit modest, to the concept that low HDL levels negatively influence the outcomes of patients following CABG. Whereas the current study focused on the process of SVG disease within the first postoperative year, LOCAT patients were enrolled years after surgery, when many had already developed SVG disease at the time of the baseline angiography. We were limited by a smaller sample size and shorter follow-up period in our study, but we found that a relationship existed between HDL levels and SVG disease using both conventional angiography as well as using more sophisticated graft imaging with IVUS. We believe our data highlights the relevance of HDL as a factor influencing the development of SVG disease, even in the current era when nearly all patients receive antiplatelet and statin therapies after surgery.

Multiple mechanisms may explain the link between HDL and the development of SVG disease. HDL particles reduce LDL oxidation and reverse cholesterol transport, promoting cholesterol efflux from macrophages and preventing foam cell development in a vessel wall [6,8]. Moreover, higher levels of HDL are profoundly anti-atherogenic and may favorably impact graft patency by preventing a procoagulant milieu [1,6]. Independent from their involvement in cholesterol metabolism, HDL also improves endothelial function and promotes endothelial repair [6]. By reducing vascular inflammation and thrombosis [6], it is possible that higher levels of HDL particles may slow the process of SVG disease, ultimately decreasing the risk of adverse outcome following surgery. Regardless of the exact mechanism of action, the biological signal demonstrated in this analysis highlights the need for further research in this area. The modulation of HDL with medical therapy may represent a valuable future strategy for the prevention of SVG disease after CABG.

The current study is the first to date to evaluate the impact of HDL levels on graft occlusion rates and vein intimal hyperplasia early after CABG. Nevertheless, the results presented must be interpreted within the context of several limitations. The CASCADE Trial was designed to assess the impact of antiplatelet therapy on the process of SVG disease 12 months after surgery [15]. Patients were not randomized to different postoperative lipid modification strategies, as these decisions were left to the discretion of physicians who were following established clinical guidelines [4]. Moreover, we acknowledge that the findings of this post-hoc analysis must be viewed as exploratory in nature. Given the modest results, we believe our findings are hypothesis-generating and require confirmation from future studies, perhaps with longer follow-up periods and larger sample sizes. Post-hoc power calculations revealed that we would have required more than 350 vein grafts in each group to have demonstrated a significant difference in the patency rate between patients who did or did not have HDL levels <40 mg/dL 12 months after surgery, assuming a statistical power of 50%.

## Conclusion

Within the CASCADE trial population, our exploratory analysis demonstrated that lower HDL levels were associated with trends towards more graft occlusions and more vein intimal hyperplasia 12 months following CABG. Patients with ideal HDL levels >60 mg/dL had significantly lower amounts of intimal hyperplasia in our cohort. Despite the limitations of a small sample size, we noted a modest signal suggesting a potential effect of low HDL levels on the development of SVG disease early after surgery. We believe that modulation of postoperative HDL levels with medical therapy may represent a valuable future strategy for the reduction of SVG disease, and further research and clinical trials appear warranted.

## Abbreviations

CABG: Coronary artery bypass graft surgery; CAD: Coronary artery disease; CASCADE: Clopidogrel after surgery for coronary artery disease; HDL: High density lipoprotein; IVUS: Intravascular ultrasound; LDL: Low density lipoprotein; LOCAT: Lopid coronary angiography trial; NS: Not significant; OR: Odds ratio; SVG: Saphenous vein graft disease.

## Competing interests

The authors declare that they have no competing interests.

## Authors’ contributions

Each author listed contributed to the development of this manuscript. Specifically, KJ helped with data analysis and drafted the paper. MR, PV, and MRLM provided patients for the subject matter of this report, established study funding, and presented critical revisions of the manuscript. AK developed the study concept, performed the statistical analysis, and drafted the manuscript. All authors read and approved the final manuscript.
